# Modulating gene expression as a strategy to investigate thyroid cancer biology

**DOI:** 10.20945/2359-4292-2024-0073

**Published:** 2024-11-06

**Authors:** Diego Claro de Mello, Joice Moraes Menezes, Antonio Tarelo Freitas de Oliveira, Marcella Maringolo Cristovão, Edna Teruko Kimura, Cesar Seigi Fuziwara

**Affiliations:** 1 Universidade de São Paulo Instituto de Ciências Biomédicas Departamento de Biologia Celular e do Desenvolvimento São Paulo SP Brasil Departamento de Biologia Celular e do Desenvolvimento, Instituto de Ciências Biomédicas, Universidade de São Paulo, São Paulo, SP, Brasil

**Keywords:** RNA interference, microRNA, CRISPR/Cas9, thyroid cancer

## Abstract

Modulating the expression of a coding or noncoding gene is a key tool in scientific research. This strategy has evolved methodologically due to advances in cloning approaches, modeling/algorithms in short hairpin RNA (shRNA) design for knockdown efficiency, and biochemical modifications in RNA synthesis, among other developments. Overall, these modifications have improved the ways to either reduce or induce the expression of a given gene with efficiency and facility for implementation in the lab. Allied with that, the existence of various human cell line models for cancer covering different histotypes and biological behaviors, especially for thyroid cancer, has helped improve the understanding of cancer biology. In this review, we cover the most frequently used current techniques for gene modulation in the thyroid cancer field, such as RNA interference (RNAi), short hairpin RNA (shRNA), and gene editing with CRISPR/Cas9 for inhibiting a target gene, and strategies to overexpress a gene, such as plasmid cloning and CRISPRa. Exploring the possibilities for gene modulation allows the improvement of the scientific quality of the studies and the integration of clinicians and basic scientists, leading to better development of translational research.

## INTRODUCTION

Modulation of gene expression, either inhibition or overexpression, is a key approach to investigate gene function. The technology of gene silencing with RNA interference is a classic and powerful tool for loss-of-function studies, but gene editing with CRISPR/Cas9 is becoming more popular as the methodology evolves. On the other hand, artificial overexpression of a gene, mainly by a plasmid-based or virus-based approach, is the main gain-of-function experiment for *in vitro* research.

In cancer research, the possibility of blocking or activating a gene has only been fruitful due to the existence of a large catalog of human cancer cell lines available, where gene modulation can be tested easily. The use of established cancer cell lines allowed consistent progress in the field, since cell lines are convenient and affordable and can replace human samples, which are not accessible to every researcher.

In this context, thyroid cancer is the most prevalent endocrine malignancy, with most cases deriving from thyroid follicular cells (1), which are responsible for converting iodine into thyroid hormones. Papillary thyroid cancer (PTC) is the most frequent form of thyroid cancer and is mostly curable with conventional treatment, while anaplastic thyroid cancer (ATC) is the rarest form, but is lethal and often refractory to therapy (2). Thus, to understand this diverse biological behavior, the establishment of cell lines was imperative, and guidelines for the appropriate care and handling of cell lines were also necessary (3).

Currently, more than 60 cell lines derived from different histological types, such as papillary, follicular, anaplastic, and medullary thyroid cancer (4), are available and widely used for thyroid cancer studies. Most advances in the thyroid field have been achieved from the integration of cancer cell line studies with *in vivo* modeling in transgenic mice and human cancer tissues, which allowed the uncovering of the main driver oncogenes and signaling pathways involved in thyroid cancer (5). For example, the transgenic mice model with selective activation of the BRAF^V600E^ mutation in the thyroid gland, which leads to thyroid cancer development and progression to more aggressive histotypes (6,7), has shown that cancer initiation may be enhanced by TSH signaling and that BRAF-induced thyroid cancer is addicted to MAPK signaling (8,9). Moreover, animal models may help the development of new cell lines derived from a patient's primary tumor (the so-called patient-derived xenograft [PDX]), which can be used to investigate a tumor's microevolution, drug response screening, validation of new drugs, and studies for drug resistance (10-12). A variation of this model is the cell line-derived xenograft (CDX), in which established cell lines are injected into immunocompromised mice and grown into tumors, also allowing for drug screening and resistance studies (13).

Recently, the evolution of high-throughput and large-scale technologies enabled the integration of human data derived from genomic, transcriptomic, and proteomic studies to be rapidly translated to *in vitro* and *in vivo* fields using cell line modifications and animals (14-16). Altogether, this cumulative knowledge has impacted clinicians’ decisions regarding surgery, drug choice, and targeted therapy in aggressive types of thyroid cancer where conventional therapy has been ineffective (17), and has collaborated with the establishment and evolution of the guidelines for undifferentiated thyroid cancer (18).

**Figure 1:**
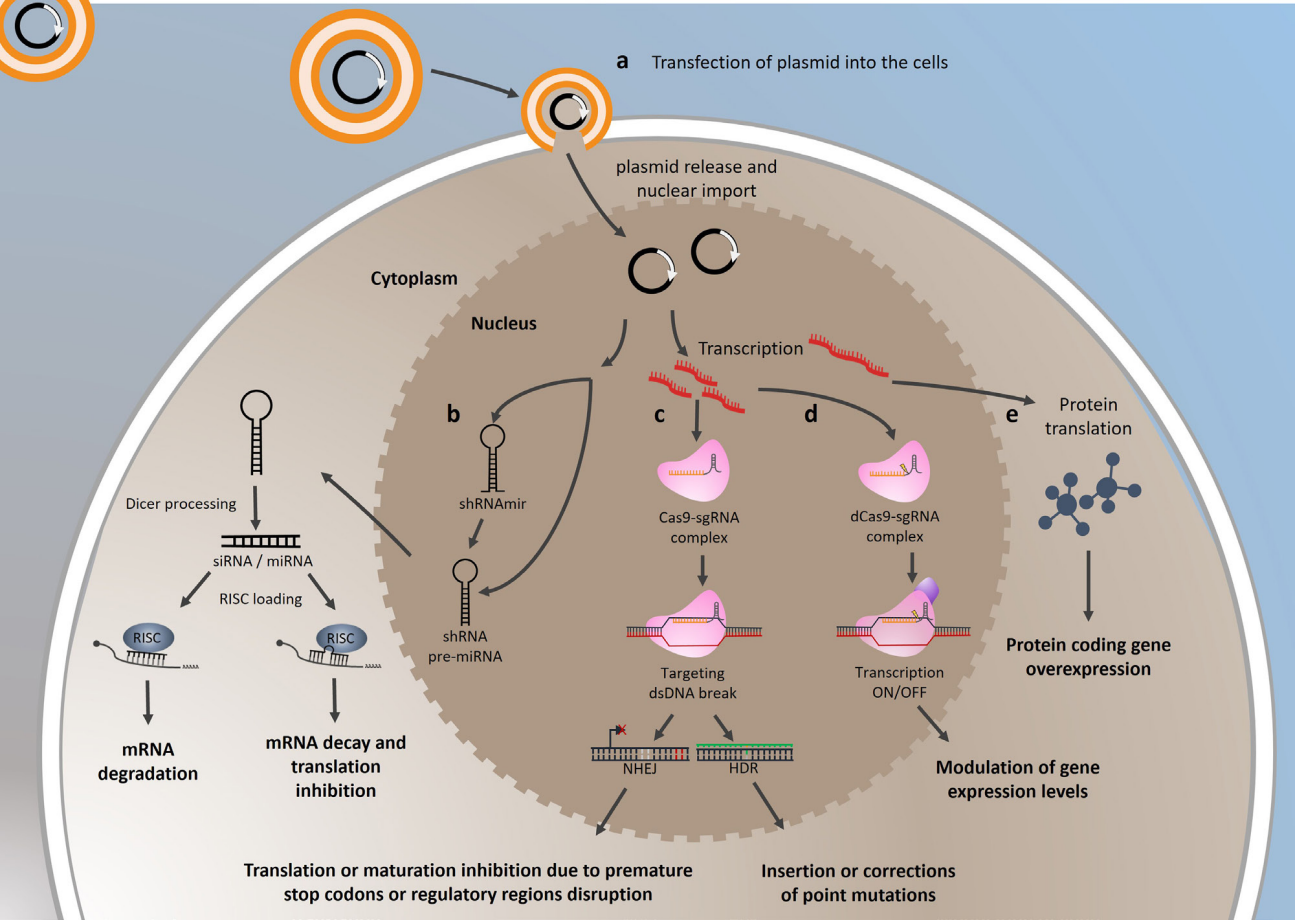
Schematic representation of the transfection process for modulating gene expression. (**a**) Plasmid is encapsulated within liposomal vesicles and delivered into cells’ cytoplasm via endocytosis. In the cell nucleus, the plasmid is transcribed into coding or noncoding RNA. (**b**) Short hairpin RNA (shRNA) or precursor microRNA (pre-miRNA) undergo maturation through nuclear processing. These transcripts are transported to the cytoplasm, where they are further processed by the Dicer enzyme to form duplex molecules of small interfering RNA (siRNA) or miRNA. When one strand of this duplex is incorporated into the RNA-induced silencing complex (RISC), it promotes mRNA degradation (siRNA) or translation inhibition (miRNA) and subsequent decay. (**c**) Single-guide RNA (sgRNA) associates with the protein Cas9 endonuclease (also transcribed by the plasmid and translated in the cytoplasm) to interact with the sgRNA target region in the genome. Upon perfect matching of the sgRNA and DNA adjacent to a PAM sequence (NGG), the Cas9 endonuclease generates a DNA double-strand break (dsDNA). Subsequently, cells undergo repair mechanisms: i) non-homologous end joining (NHEJ) repair, which may introduce insertions or deletions (indels) in the original DNA, disrupting translation via the presence of premature stop codons or affecting noncoding RNAs maturation and ii) homologous-directed repair (HDR), involving a DNA-template donor. Following the dsDNA break, cells utilize the template, often with a single nucleotide modification, to generate point mutations or insertions. (**d**) sgRNA interacts with a catalytically inactive Cas9 (dCas9) and targets specific DNA regions to recruit proteins that either induce or inhibit transcription of the target gene. (**e**) The coding sequence of a protein-coding gene is transcribed into mRNA which is subsequently translated into functional proteins.

Adapted from: Shalem O, Sanjana NE, Zhang F. High-throughput functional genomics using CRISPR-Cas9. Nat Rev Genet. 2015;16(5):299-311. doi: 10.1038/nrg3899.

In this review, we cover the main approaches to modulate gene expression *in vitro* using cell lines for cancer investigation, including strategies to inhibit gene expression through RNA interference, CRISPR/Cas9, and dCas9 and to overexpress protein-coding genes and noncoding genes (Figure 1). We hope this review encourages the implementation of these techniques, which are currently much more accessible and may enrich and improve both basic and translational research in the field of thyroid cancer.

### Strategies to reduce a target gene expression

#### Reducing coding gene expression

One of the most classic tools for downregulating gene expression is RNA interference (RNAi). Discovered by chance in a plant study that aimed to increase the color of petunia flowers by overexpressing the gene Chalcone synthase (*CHS*) but resulted in a loss of color (19), and later described in *C. elegans* (20), RNAi remains currently popular with some improvements in the generation of stable knockdown systems.

The RNAi process involves targeting a gene that is knocked down with high specificity and selectivity using an antisense small RNA. The gene silencing occurs as a result of RNA degradation into short RNAs, which then activate ribonucleases to target corresponding mRNA (21). The targeted induction of RNAi can be achieved in two different ways: chemically synthesized double-stranded RNA (dsRNA)-small interfering RNA (siRNA) and vector-based short hairpin RNA (shRNA) (22). Despite them having comparable functional outcomes with mRNA knockdown, it is imperative to recognize the intrinsic differences between siRNA and shRNA molecules.

**Figure 2:**
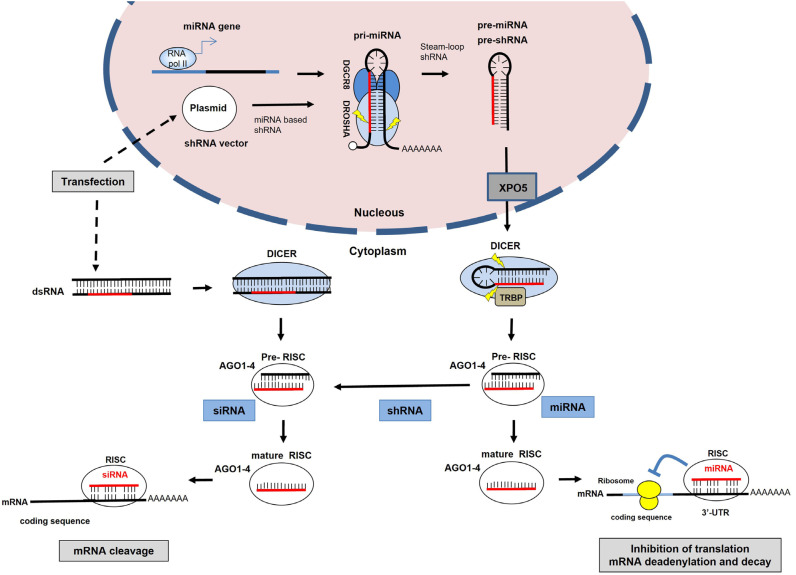
The RNA interference (RNAi) pathway uses endogenous miRNA processing machinery components. This pathway is guided by small interfering RNA (siRNA) generated from exogenous double-stranded RNA (dsRNA) molecules or from shRNA structures, inducing the silencing of target mRNA. As with endogenous miRNAs, synthetic miRNA-based shRNAs (plasmid) are expressed from RNA polymerase II (Pol II) promoters as primary miRNA transcripts (pri-miRNAs). The complex DROSHA/DGCR8 performs cleavage of pri-miRNA and gives rise to a miRNA precursor (pre-miRNA), resulting in the generation of a pre-miRNA. Following export to the cytoplasm, the pre-miRNA undergoes additional processing by the DICER endonuclease. The resultant miRNA duplex associates with the RNA-induced silencing complex (RISC), where the mature strand of the miRNA is retained. This RISC-microRNA complex engages in imperfect binding to the 3’-UTR regions of target mRNA, initiating a reduction in protein levels through mechanisms such as translation blockage, mRNA deadenylation, and decay. In the RNAi pathway, the resulting siRNA produced by DICER cleavage of pre-shRNA or long dsRNA is incorporated into Argonaute 2 (AGO2). If the RNA duplex loaded onto RNAi-induced silencing complex (RISC) has perfect sequence complementarity, AGO2 cleaves the passenger (sense) strand so that active RISC containing the guide (antisense) strand is produced. The siRNA guide strand recognizes target sites to direct mRNA cleavage (which is carried out by AGO2).

The RNAi function cross-talks with an endogenous pathway utilized by microRNAs (miRNAs) for posttranscriptional control of gene expression (Figure 2). Indeed, the efficacy of RNAi is dependent on the miRNA processing machinery (23). In the endogenous pathway, RNAs containing short hairpin structures (such as miRNAs) are processed in the nucleus and exported to the cytoplasm in the form of precursor molecules identified as precursor miRNAs (pre-miRNAs) (24). In the cytoplasm, pre-miRNA undergoes further processing by the RNAse III enzyme Dicer to produce an imperfectly matched double-stranded miRNA. Dicer performs a similar processing function on long, perfectly matched dsRNA, which is the most usual form of delivering synthetic siRNA to the cells. The dsRNA is cleaved by Dicer, yielding siRNA intracellularly, which becomes a ∼21nt dsRNA (23). A multienzyme complex, which includes Argonaute 2 (AGO2) and the RNA-induced silencing complex (RISC), binds to the miRNA duplex or siRNA duplex and this interaction leads to the expulsion of one strand to form an activated complex containing the antisense strand (25). In the RNAi pathway, the activated AGO2-RISC-siRNA complex then binds to perfectly complementary sequences in the mRNA (usually in the coding region), leading to the cleavage of the mRNA and degradation. On the other side, the AGO2-RISC-miRNA complex binds to the 3’-untranslated region (UTR) of the target mRNA to block the translation.

Currently, small interfering RNAs (siRNAs) are available from different companies and are usually delivered as a pool of three different siRNAs (initially as dsRNA), targeting different segments of the coding region of the mRNA. This strategy is important to ensure a considerable knockdown of the target gene. Indeed, the combination of multiple siRNAs targeting the same gene increases knockdown efficiency to >70%, in theory (26). Nevertheless, the knockdown effect of siRNA is transient and usually ceases after 5-7 days in cancer cell lines that are constantly dividing (26).

In this context, the use of a shRNA plasmid-based approach is preferred to achieve a stable knockdown, as it will lead to endogenous production of the shRNA that will be cleaved into siRNA (Figure 2). Following the delivery of a shRNA expression vector into the cytoplasm by transfection, for example, subsequent transport for the nucleus is essential for transcription, which usually occurs after mitosis and nuclear envelope reorganization (27). Primary transcripts containing the shRNA (pri-shRNA) are transcribed and follow a similar route to that discovered for miRNA (explored later in this section) (28). These primary transcripts undergo processing facilitated by the Drosha/DGCR8 complex, giving rise to precursor shRNAs (pre-shRNA). Subsequently, pre-shRNAs are transported to the cytoplasm through exportin 5, where they are further processed by Dicer, culminating in the maturation of the shRNA into siRNA. The mature siRNA bound to Dicer associates with the RISC and RNAi is triggered when there is a perfect complementarity of siRNA and target mRNA, leading to its cleavage by AGO2 and subsequent degradation (23).

In order to improve endogenous shRNA production from an expressing vector, the use of a conserved miRNA segment known to be efficiently processed by Drosha/Dicer fused to the shRNA sequence has shown enhanced gene knockdown results. This technique is classified as a shRNAmiR structure and shows some advantages over regular shRNA modulation, which consists of a double-stranded RNA molecule with a hairpin structure (29,30). The main advantages are: (A) the potential for the shRNAmiR to be expressed in a tissue-specific manner since it can be transcribed from a Pol II promoter like the miRs in the cell; (B) a smaller probability of inducing general cell toxicity, as it can be regulated by Pol II cell-specific promoters; and (C) small number of off-targets, since Dicer (miRNA biogenesis processing enzyme) cuts the shRNAmiR molecule precisely when it recognizes a loop, which is present in the miRNA backbone structure, efficiently generating siRNAs (30).

One successful modification named miR-E technology optimized the backbone structure of miR-30 by fusing it with the shRNA sequence and introducing some modifications for Dicer to retain the 5p strand as the antisense sequence (29). This modification produces primary shRNA structures with enhanced processing, boosting the levels of siRNA and generating a consistent and stronger gene knockdown. To design the shRNA sequence in the stem structure of miR-30 backbone, the program splashRNA (http://splashrna.mskcc.org/) was developed, which uses a sequential learning algorithm to generate a list of guide sequences of shRNAs, based on the coding sequence of the target gene as input (31). Then, the program creates the 97-mer oligonucleotide, which contains the shRNA sequence in a functional structure with the miR-E backbone. This fragment, ordered as an oligonucleotide, is then amplified by PCR using specifically designed mir-E primers to insert the cloning enzymes in the fragment to be cloned in the plasmid sGEN (green fluorescence protein [GFP] + neomycin resistance), for example. After that, the resulting plasmid is transfected or transduced as viral particles that deliver the DNA segment into the cell nucleus, where, after DNA integration (for viruses), it leads to the transcription of miR-like hairpin structure (Figure 2). These hairpins are processed by endogenous miRNA machinery to yield siRNA to be loaded into the RISC complex resulting in target gene knockdown by RNAi mechanism.

### Reducing noncoding gene expression

#### microRNAs

As already mentioned at the beginning of this section, the mechanism of action of the RNAi (either using siRNA or shRNAs) relies on the existence of the endogenous miRNA machinery (Figure 2). MiRNAs are small noncoding RNAs (∼18-23 nucleotides [nt]) that regulate gene expression post-transcriptionally mainly by interacting with the 3’- UTR region of the mRNA (28). The primary mechanism is associated with translation modulation as miRNAs may bind imperfectly to the 3’-UTR of mRNAs and impair the translation process either by blocking the translation or inducing mRNA decay (32). This characteristic imperfect pairing allows the regulation of hundreds of targets simultaneously and creates a complex miRNA-regulatory network inside the cells.

Since the discovery of the first miRNA in humans, the miRNA let-7 (33), more than 2,600 mature miRNAs have been identified (34), and an exponential amount of evidence has been published to reinforce the role of miRNAs in cancer. The association between miRNA deregulation and cancer was initially observed in chronic lymphocytic leukemia (35) and lung cancer (36), and the subsequent validation of oncogenes such as *BCL2* and *RAS* as miRNA targets. Deregulation of miRNA expression is frequently observed in cancer and is a potent marker for malignancy and prognosis (37-39).

In thyroid cancer, a pioneer study showed the upregulation of several miRNAs in PTC (40), including the first report of miR-146b-5p. After almost 20 years of studies using high throughput methodologies (14,41), cell lines (42,43), and clinical data (44,45), miR-146b became a consolidated oncomiR in thyroid cancer. It is interesting to note that a single miRNA such as miR-146b-5p may interfere with a variety of cellular processes, such as cell differentiation, proliferation, migration, and invasion (42,43,46-49), enhancing the complexity of the miRNA-regulatory network in cancer.

In order to investigate the role of an overexpressed miRNA such as miR-146b, loss-of-function assays are employed mostly using a temporary approach with antisense oligonucleotides (ASO), artificially synthesized oligonucleotides complementary to mature miRNA that block the miRNA function and lead to derepression of miRNA targets.

Notably, miRNA-targeting ASOs – also known as antimiR or antagomiR – promote silencing by strongly binding miRNAs and causing steric blocking (50). AntimiR molecules contain chemical modifications, such as locked nucleic acid (LNA) (51), optimized to improve their stability, binding affinity, and inhibitory effect on the target miRNA (50,52). The preferable method for antimiR delivery into cells is transfection, and similarly to siRNA, the effects of antimiR are temporary and usually reduced after some days as cells divide.

For a stable blockage of miRNA function, the design of miRNA sponges may be used as another strategy. In this method, a transgene harboring multiple miRNA target sites in the 3’-UTR region is overexpressed and sequesters mature miRNA by competition (53), leading to the derepression of miRNA-targeting mRNAs. For the construction of a miRNA sponge, the design of a miRNA target complementary sequence is essential, as described by Kluiver and cols. (54). Then, the insert, which is ordered as a pair of oligonucleotides containing two repeats of the anti-miRNA sequence and sites for SanDI in both ends, is cloned into a modified recipient plasmid that contains a strong promoter region upstream the cloning site with the SanDI and a selection marker. The SanDI site cloning allows the concatemerization of several anti-miRNA fragments and creates a sponge with up to hundreds of repeats. As this is a stable approach, cell lines transfected with the sponge may be injected into immunocompromised mice to evaluate the effect of miRNA blockage on tumor growth *in vivo*.

#### Long noncoding RNAs

The long noncoding RNAs (lncRNAs) are another class of noncoding RNAs classified as larger than 200 nt in size, which includes very large lncRNAs such as X-inactive specific transcript (XIST) and Air, with ∼17 Kb and 108 Kb, respectively. The estimated number of lncRNAs is currently 96,308 (55), exceeding the number of coding genes in the Telomere-to-Telomere (T2T) Consortium (*i.e.*, 19,969) (56). However, recently, due to the wide variety of noncoding RNA species identified around this size range, such as RNA polymerase III transcripts as well as small nuclear RNAs (snRNAs) and some small nucleolar RNAs (snoRNAs), it has been proposed to classify them as noncoding RNAs (ncRNAs) with more than 500 nt, usually transcribed by RNA polymerase II (57).

Much about the mechanism of action of lncRNAs is still poorly understood; however, their biological functions are broader than those of miRNAs (58). Due to its larger size, lncRNA may form structures that interact with DNA, RNA, and protein and may participate in the regulation of gene expression at multiple levels such as (A) in the control of chromatin structure, by recruiting protein complexes associated with histone modifications; (B) in the structure of many nuclear ribonucleoprotein domains, as well as of cytoplasmic membraneless organelles; (C) in the modulation and fine-tuning of gene expression at the transcriptional level through *cis* action; and (D) in the regulation of miRNA activity as competing endogenous RNAs (ceRNAs), acting as natural sponges by binding miRNAs (59).

Knocking down lncRNAs with RNAi is feasible and sometimes challenging, depending on the size of the lncRNA and its regulatory structural domains. Targeting the 2346 bp long SNHG3 lncRNA with shRNA results in strong knockdown (∼80%) in gastric cancer, for example (60). On the other hand, targeting the ∼17 Kb lncRNA XIST results in ∼50%-60% knockdown in breast cancer cell lines (61). Importantly, the cellular localization of the target lncRNA should be considered when selecting silencing methodologies. Notably, RNAi was reported to be more effective at silencing cytoplasmic lncRNAs, but less effective against nuclear localized lncRNAs when compared with RNAse H1 activity-inducing ASOs (62).

### Gene editing with CRISPR/Cas9 for coding and noncoding genes

Another strategy for loss-of-function studies is the use of gene editing methodologies to alter the DNA sequence and impair target gene transcription/translation. In the recent past, gene editing with TALEN or ZFN was usually chosen as the last option due to several methodological difficulties, but with the advent of CRISPR/Cas9, gene editing has become the first option for gene modulation at the DNA level (63).

**C**lustered **R**egularly **I**nterspaced **S**hort **P**alindromic **R**epeats, commonly known as CRISPR, is a revolutionary tool for genetic engineering that has transformed the landscape of gene expression modulation. The recognition came in 2020 with the Nobel Prize in Chemistry for the remarkable reprogrammability of CRISPR/Cas9 to precisely edit the DNA (64,65), and the potential applications of this tool in several fields of research (66).

At its core, CRISPR/Cas9 is inspired by the adaptive immune system of bacteria, particularly their defense mechanism against viral infections. This adaptive immune system allows bacteria to recognize and target specific viruses that have previously invaded their cellular machinery. The infection by bacteriophages (viruses that target bacteria) may deploy a defense mechanism. This system involves capturing and storing fragments of the viral DNA within the bacterial genome in the form of CRISPR sequences (67). These stored sequences are transcribed into CRISPR RNA (crRNA), which interacts with the trans-activating CRISPR RNA (tracrRNA) to be loaded into Cas9 protein to serve as effectors of adaptive immunity. When the same or a closely related virus reinfects the bacteria, the complex crRNA/tracrRNA+Cas9 can detect the foreign DNA due to crRNA complementarity, and the Cas9 complex works as a molecular scissor to target precisely and cleave the viral DNA.

The Cas9 endonuclease is a crucial component of the CRISPR/Cas9 system as it functions by introducing double-strand breaks into the target DNA. The two nuclease domains of Cas9 protein – RuvC and HNH – play distinct roles in this process. Briefly, the RuvC domain is responsible for cleaving the non-target strand of the DNA, while the HNH domain is responsible for cleaving the target strand of the DNA. Both domains possess endonuclease activity, inducing a break in the phosphodiester backbone of the targeted DNA strand (66).

Over the course of a decade, CRISPR/Cas9 has evolved into a groundbreaking tool for precise gene modulation. One key advancement came with the fusion of guide RNA (gRNA) and tracrRNA into a single structure named single-guide RNA (sgRNA) by pioneers Charpentier and Doudna (64). This modification enhanced the tool's programmability, facilitating the cloning of the gRNA sequence and enabling the precise targeting of almost any genomic region.

The sgRNA contains a 20-nucleotide segment (the programmable fragment) that is complementary to the target site in the genome adjacent to an NGG sequence (PAM sequence). When a perfect match of sgRNA sequence and target DNA occurs, the Cas9 endonuclease cuts the double-stranded DNA (dsDNA), and the endogenous DNA repair system performs the gene editing (66). The highly efficacious non-homologous end joining (NHEJ) is the preferable mechanism to ligate the DNA broken ends but may result in random insertion and deletions due to the lack of a template DNA (66). The consequence of NHEJ is the disruption of gene function by changing the original reading frame, leading to protein truncation, insertion of premature stop codons, loss of function, or degradation. As an alternative to NHEJ repair, cells can undergo homologous direct repair (HDR) by using a DNA donor template. The HDR methodology can be used to cause or correct point mutations in the DNA and is a powerful tool for the understanding of cancer biology and genetics (66).

It is worth noting that for eukaryotic cells, the Zhang Lab introduced strategies that further expanded the application of CRISPR/Cas9 in more complex organisms (68). For that, the group showed that CRISPR/Cas9 is suitable in mammals such as humans and mice by delivering a single plasmid that contains cloning sites for the sgRNA and encodes for Cas9 endonuclease and a selection mark. This strategic adaptation facilitated a broad range of applications in gene modulation, enhancing the versatility of CRISPR/Cas9 technology in different fields of study.

Protein-coding genes are the primary focus in CRISPR/Cas9 gene editing due to the higher probability of generating a change in the reading frame that could lead to loss of protein levels. For that, a variety of online tools, such as CHOPCHOP, Gene Perturbation Platform, and CRISPick (69,70) facilitate the design of specific sgRNAs, predicting their efficiency in DNA cleavage and aiding researchers in the selection process. If a protein with loss of function is desired, sgRNAs flanking the first exons or near the start codon site (*ATG*) are recommended (68).

Once the desired sgRNA is chosen, a common and cost-effective method for implementing CRISPR-mediated gene editing involves cloning of sgRNA into expression vectors (71). For example, the simplest plasmid for this system is the plasmid PX459 (Plasmid #48139), which is available in the Addgene database, a plasmid deposit (72). This plasmid contains the cloning site for the desired sgRNA and expresses Cas9 and a puromycin resistance marker. The cloning is performed by annealing oligonucleotides to create cohesive ends to be ligated into BbsI-digested plasmids (71). These vectors carry the coding sequence for Cas9 and a selection marker, often using puromycin or GFP. Next, the vector is transfected (or transduced, in the case of lentiviruses) into cells and then selected with the antibiotic or cell sorted according to their fluorescence (68,71). The validation of coding gene editing is performed through protein analysis, such as Western blotting, which should show a loss of protein expression, and by DNA sequencing of the CRISPR/Cas9 target region. After confirming the loss of function of the CRISPR/Cas9-targeted gene, cells are suitable for subsequent experimental investigations.

Thyroidologists have benefited from the application of CRISPR/Cas9 in cell line studies investigating thyroid cancer, whose publications were recently compiled in a review (73). In this context, gene editing with CRISPR/Cas9 has identified potential molecular targets for treatment and potential biomarkers for use in thyroid cancer research, such as EZH2 (74), HMGA2 (75), and GPX4 (76).

Recently, our group investigated the effect of EZH2 inhibition with CRISPR/Cas9 and a selective inhibitor EPZ6438 in ATC cells (74). Notably, EZH2 is the catalytic subunit of the Polycomb Repressive Complex 2, an epigenetic regulator of chromatin accessibility (77). Overexpression of EZH2 has been identified in thyroid cancer, especially in ATC, and its inhibition results in antitumoral effects *in vitro* and *in vivo*, reducing cell growth, migration, and invasion and improving differentiation of ATC cells (74,78). Interestingly, mutual inhibition of EZH2 and HMGA, a group of architectural chromatin proteins that are overexpressed in thyroid cancer, significantly increased apoptotic rates and induced cell cycle arrest in ATC cells (79). Moreover, a recent study showed that *HMGA2*-mediated gene editing with CRISPR/Cas9 impaired cell growth and migration *in vitro* in PTC cells (75). Together, these findings reinforce the importance of epigenetics in thyroid cancer biology and aggressiveness.

Recently, a CRISPR screening approach was used to target differentially expressed genes in oncocytic thyroid carcinoma characterized by mitochondrial alterations and DNA mutations (76). CRISPR screening is a high-throughput approach that uses several tagged sgRNAs to target different genes in one pooled sample (80) in order to identify which gene knockout reverts a phenotype. In this work, it was found that the *GPX4* gene maintains the mitochondrial disorders in oncocytic thyroid carcinoma, whereas its inhibition confers vulnerability to this cancer type (76).

Targeting ncRNAs with CRISPR/Cas9 is also possible and has become a long-term efficient and feasible method compared with RNAi for ncRNAs (81). Similar to the process for protein-coding genes, the design of sgRNAs for ncRNAs can be easily done by web tools such as CHOPCHOP (70). However, for more efficient results in disrupting gene function, the sgRNA should target regulatory or structural regions of the ncRNA gene (73,82). For instance, for miRNAs-mediated gene editing, Drosha or Dicer processing sites or the principal strand (5p or 3p) targeting is desired (28,73). Our group has shown that editing a miRNA gene such as *MIR146B* or a cluster *MIR17-92*, both overexpressed in thyroid cancer, is possible using the CRISPR/Cas9n system that uses two sgRNA targeting opposite strands of the target DNA in close proximity to generate a dsDNA break (83,84). The resulting gene editing leads to a reduction of mature miRNA expression levels due to a loss of regulatory segments within the precursor structure and impaired processing.

Editing lncRNAs with CRISPR/Cas9 is also possible, but one of the main problems is that lncRNA may be a very long RNA, and editing the DNA may just remove some fragments of the lncRNA sequence, not fully blunting its function as protein scaffolds or miRNA sponges (85). By targeting regulatory regions of the lncRNA, more efficient results may be achieved in disrupting a lncRNA gene function (73,82). For example, human XIST is a 17 Kb lncRNA that presents several tandem repeats, named A-F, that are involved in XIST expression and regulation (86). Targeting the D-repeat using a sgRNA effectively reduces the expression of XIST and derepresses the expression of X-linked genes, indicating that this region is essential for X-chromosome inactivation by XIST (82). Moreover, it should be noted that about two thirds of all annotated lncRNA sequences are located in close proximity to, or overlap with, protein-coding or other noncoding RNAs in the human genome (85), which could lead to potential side effects (such as also disrupting a coding gene) that should be properly investigated when validating the extension of gene editing by sequencing.

The CRISPRi (CRISPR interference) is a variation of the CRISPR/Cas9 system used to inhibit a gene expression without inducing gene editing. The CRISPRi system employs dead Cas9 (dCas9), which contains mutations in the RuvC and HNH domain, rendering dCas9 unable to cut the DNA. Nevertheless, dCas9 maintains the ability to interact with sgRNA and bind to target DNA, and this property is used to engineer a chimeric dCas9 protein fused with the active domain of chromatin modifiers such as the Krüppel-associated box (KRAB). In this system, the dCas9-KRAB inhibits the target gene expression by blocking RNA polymerase assembly and function when the sgRNA is directed to the start codon or proximal promoter region of the gene (87).

### Strategies to increase a target gene expression

**Figure 3:**
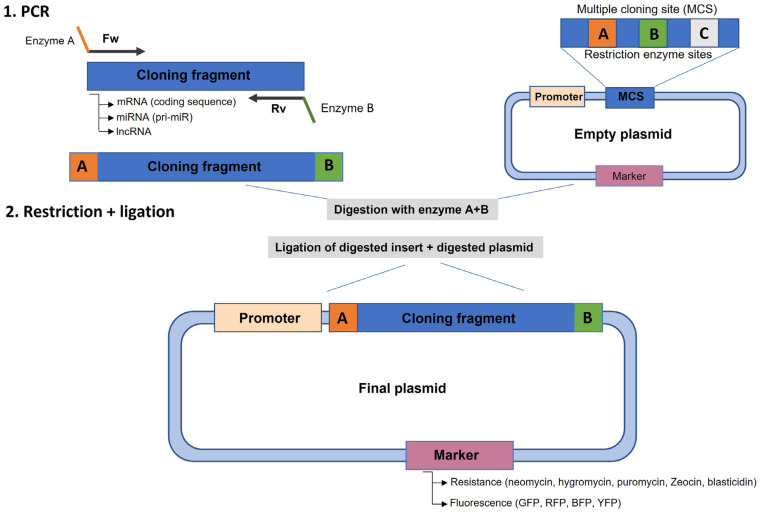
Scheme for PCR cloning a target gene into an expression vector. **1.** PCR amplification of the target fragment (cDNA, miRNA, lncRNA, etc.) with primers that add restriction enzyme sites to the amplicon that are compatible with the enzymes present in the multiple cloning site (MCS) of the plasmid. **2.** Digestion of the PCR fragment and plasmid with both enzymes and ligation with T4 ligase to create the final plasmid that contains the insert cloned downstream of a promoter and a selection marker, either antibiotic resistance or fluorescent marker for screening in the target cell line.

To overexpress a coding gene, it is first necessary to understand the gene structure, localization, and – most importantly – the mRNA characteristics. Usually, the cloning process involves the identification of the size of the coding sequence (CDS) from the start codon to the stop codon (88). Depending on the size of the CDS, amplification by PCR may be challenging and demand time for optimization. A conventional and easier cloning method relies on the introduction of two different restriction enzyme sites in the 5’ and 3’ regions of the amplicon using modified primers (Figure 3). It is important to note that these enzyme sites should not occur inside the amplicon sequence to avoid cloning truncation. Moreover, depending on the recipient plasmid chosen (for example, a tag plasmid), the stop codon should be removed to allow the translation of the protein tag in the C-terminal region of the protein. Next, the plasmid should possess a strong promoter region – such as MSCV, EIFα, or PGK – driving the transgene expression (89). It is also important to add the Kozak sequence before the first codons of the transgene to ensure that the protein is translated (90), although this step may be omitted for some types of promoters.

To overexpress a miRNA, it is necessary to understand its structure and how it is transcribed and processed by cells. The miRNAs are transcribed by RNA polymerase II in long primary transcripts (pri-miRNA) that are processed by the microprocessor complex (Drosha+DGCR8) into pre-miRNA, which presents a hairpin conformation. Next, the pre-miRNA is further processed by Dicer into a miRNA duplex that enters the RISC complex to block protein translation (28).

Determining the genomic structure of a miRNA gene is complex. For years, knowing the mature and precursor miRNA sequence was the only characteristic that was consolidated in the literature and in the database (34), as the primary sequence could reach Kb in length. Indeed, the Functional Annotation of the Mammalian Genome (FANTOM5) project used the cap analysis of gene expression (CAGE) methodology to identify the transcription start site (TSS) of miRNA genes and delimit its promoter regions (91). This project revealed that pri-miRNA sequences are usually long RNA segments, for example, the pri-miRNA for the cluster *MIRLET7FA/MIRLET7F* is 34.1 Kb long.

In this sense, cloning the primary sequence of some miRNAs would not be feasible methodologically. Thus, cloning the precursor miRNA is a convenient alternative as it has a hairpin conformation with ∼80 nt in length. The information about the precursor sequence can be retrieved from miRBase (http://mirbase.org/) (34). In order to maintain the regulatory elements for the correct processing by miRNA machinery (Drosha/Dicer), it is recommended to amplify a ∼200 nt flanking region downstream and upstream the precursor sequence, leading to a total of ∼500 nt sequence for cloning in the plasmid.

Next, the simplest way is to choose a conventional plasmid with a selection marker, either antibiotic or fluorescence, such as MSCV Puro (puromycin resistance), and clone the amplified miRNA segment according to the restriction enzymes that were chosen in the plasmid, usually using two different restriction enzymes present at the multiple cloning site of the plasmid.

For a long noncoding RNA, whose size may range from 500 nt to 108 Kb (lncRNA Air), the cloning strategy will be evaluated individually, as plasmid cloning capacity is limited. In those cases where the cloning capacity is a problem due to longer lncRNA sequences, such as that of XIST, with over 17 Kb in length, alternative cloning vectors have been used, such as yeast artificial chromosomes (YAC) (92) and bacterial artificial chromosomes (BAC) (93).

Another strategy to overexpress a gene, including those genes with cloning issues such as longer lncRNAs, is the CRISPRa (activation) system. As described in the previous section "Gene editing with CRISPR/Cas9 for coding and noncoding genes," dCas9 may be reengineered to be fused with chromatin remodelers, which includes activators that, when interacting with a target DNA sequence by means of sgRNA, may trigger chromatin changes that induce DNA transcription (73). One of the most effective systems is the dCas9-VP64, where dCas9 is fused to VP64 (tetrameric repeat of VP16 from herpes virus) that activates transcription when the complex sgRNA+dCas9-VP64 is targeted to the proximal promoter region (87).

### Perspectives

Gene modulation methodologies have never been so accessible to researchers, and with the advent of CRISPR/Cas9, gene editing is becoming a reality for *in vivo* uses. Advances in the chemistry field improved miRNA, siRNA, and shRNA synthesis, enhanced delivery methods, and reduced immune response to accelerate *in vivo* applications as potential therapy (94).

Several clinical trials are investigating the safety of gene modulation *in vivo*. For example, miRNA-based therapies are under testing, either aiming to restore the expression of miRNA that is downregulated or to block the expression of a miRNA that is upregulated (95). One of the most promising trials is the blocking of miR-122 interaction with hepatitis C virus (HCV) using antimiR-122 (Miravirsen), which is already being evaluated in a phase 2 clinical trial for the treatment of HCV (96). In the gene editing field, CRISPR/Cas9 has been used for cancer immunotherapy, where a patient's T cells are extracted and modified *in vitro* to target cancer cells in a methodology called chimeric antigen receptor-T (CAR-T) (97). Currently, seven CART-T therapies are approved by the FDA for the treatment of blood cancers, indicating a promising future for this therapy.

While *in vivo* therapeutic applications for gene modulation are under trial, exploring the effects of gene modulation *in vitro* is still mandatory for studying cell effects and improving safety and specificity. In this process, the integration of basic scientists and clinicians is necessary, as these methodologies evolve fast and become widely diffused for the population.
